# The Impact of Anxiety and Depression on Health-Related Quality of Life in Hyperlipidemic Adults in the United States

**DOI:** 10.3390/jcm14020370

**Published:** 2025-01-09

**Authors:** Monira Alwhaibi

**Affiliations:** 1 Department of Clinical Pharmacy, College of Pharmacy, King Saud University, Riyadh 11149, Saudi Arabia; malwhaibi@ksu.edu.sa; 2 Medication Safety Research Chair, College of Pharmacy, King Saud University, Riyadh 11149, Saudi Arabia

**Keywords:** anxiety, hyperlipidemia, depression, quality of life, RAND-12

## Abstract

**Background:** Mental health issues can significantly affect the health-related quality of life (HRQoL) of adults suffering from hyperlipidemia. Therefore, in this study, the aim was to examine how depression and anxiety are related to the HRQoL of adults with hyperlipidemia. **Methods:** Data from the Medical Expenditure Panel Survey for 2016 through 2022 were used to identify adult patients diagnosed with hyperlipidemia aged 18 or older. The RAND-12 Physical and Mental Component Summary (PCS and MCS) was used to determine HRQoL. After considering variables such as age, gender, socioeconomic status, and comorbidities, linear regression was used to investigate the relationship between anxiety, depression, and HRQoL in individuals with hyperlipidemia. **Results:** A sample of 7984 adults with hyperlipidemia was identified; 9.0% experienced depression, 10.2% had anxiety, and 6.8% had both disorders. The HRQoL mean scores were lowest for adults with depression and anxiety compared to those with hyperlipidemia only. Results from the adjusted linear regression analysis revealed that hyperlipidemia patients with depression (MCS: β = −5.535, *p*-value < 0.0001), anxiety (MCS: β = −4.406, *p*-value < 0.0001), and both depression and anxiety (MCS: β = −8.730, *p*-value < 0.0001) had a significantly lower HRQoL compared to patients with hyperlipidemia only. However, in this study, it was also found that those who were physically active and employed had notably higher scores on the PCS and MCS than those who were not. **Conclusions:** The links between anxiety, depression, and lower HRQoL in patients with hyperlipidemia are clarified in this nationally representative study. This research also revealed the adverse effects of coexisting chronic conditions on HRQoL while emphasizing the benefits of employment and regular exercise. Importantly, these findings provide a compelling case for enhancing healthcare planning, allocating resources, and promoting lifestyle changes in adults with hyperlipidemia, underlining the importance of addressing mental health issues in this population.

## 1. Introduction

Health-related quality of life is an important outcome measure for individuals with chronic illnesses such as hyperlipidemia. It encompasses all the various dimensions of an individual’s physical, mental, and social well-being [1]. Health-related quality of life (HRQoL) can be negatively impacted by hyperlipidemia and other chronic illnesses. Hyperlipidemia is a group of inherited and acquired conditions that are characterized by an elevated level of lipids (fats) in the blood, which includes cholesterol, triglycerides, or both [2]. Hyperlipidemia can be divided into the two categories of a genetic condition known as primary hyperlipidemia that can result from abnormalities in a number of proteins that are involved in lipid transport or metabolism [3], and a secondary hyperlipidemia that can result from changes in lipid metabolism brought on by a variety of illnesses or factors, including endocrine disorders (like diabetes mellitus), liver diseases (like cirrhosis), renal diseases (like nephrotic syndrome), dietary factors (like a high-fat diet), or medications (like corticosteroids).

It is usually a chronic, progressive illness that requires dietary and lifestyle modifications as well as that it might be necessary for additional lipid-lowering drugs. Around 12% of adults in the United States (US) have hyperlipidemia in general [4]. It is estimated that less than 35% of those individuals effectively manage their hyperlipidemia, indicating an illness not receiving enough treatment [2]. Untreated or undertreated hyperlipidemia can lead to many forms of vascular diseases, such as peripheral artery disease, coronary artery disease, cerebrovascular accidents, type II diabetes, and hypertension [2]. The consequences of hyperlipidemia on a person’s health can be significant and financially burdensome [5,6]. Hyperlipidemia was one of the ten most costly medical conditions in the US adult population in 2008 [7]. It can significantly impact people’s psychological well-being; individuals with hyperlipidemia are at high risk of depression, according to epidemiological studies that found a bi-directional relationship between hyperlipidemia and depression [8,9,10]. In addition, hyperlipidemia is significantly related to HRQoL which in turn is related to the hyperlipidemia patients’ health outcomes.

Therefore, maintaining a high HRQoL in the face of the problems caused by hyperlipidemia may be one of the main goals for adults with hyperlipidemia. This could be achieved by lipid control, adherence to medications, and examining the psychological health of adults with hyperlipidemia. For example, evidence from a study comparing the HRQoL of individuals who experience different levels of lipid control supports that controlled lipid profiles were positively related to HRQOL [11,12]. In addition, medication adherence was found to be independently correlated with a high HRQoL score in hyperlipidemia [13,14]. However, depression had a significantly lower HRQoL as evidenced by a study in China among 10,115 hyperlipidemia patients [15]. There are few studies that have looked at the relationship between depression, anxiety, and HRQoL in adults with hyperlipidemia [15]. Therefore, understanding the causes underlying a lower HRQoL is essential, especially when considering mental health. We hypothesized that anxiety and depression are associated with lower HRQoL in adults with hyperlipidemia.

## 2. Methods

### 2.1. Study Design and Data

A retrospective longitudinal study design was used. Data were retrieved from the Medical Expenditure Panel Survey (MEPS) for 2016 to 2022. MEPS is a nationwide survey that gathers information from United States (US) citizens on demographics, socioeconomic status, insurance, medical conditions, medication usage, healthcare utilization, cost, etc.

### 2.2. Inclusion and Exclusion Criteria

In this study, adults were included aged between 18 and 64 years old who were diagnosed with hyperlipidemia. Adults must have no missing data on HRQoL and be alive within the study years. To identify adults with hyperlipidemia from MEPS data, we used the International Classification of Diseases, tenth revision, Clinical Modification (ICD-10-CM) clinical diagnosis codes (E78) from the MEPS Medical Condition File data [16]. We excluded adults with missing data on HRQoL and those without a diagnosis of hyperlipidemia.

### 2.3. Measures

#### 2.3.1. Outcome: Health-Related Quality of Life (HRQoL)

MEPS evaluated the HRQoL using the Veterans RAND 12 Item Health Survey (VR-12©) [17]. It is a self-administered health survey composed of 12 items that evaluate 8 health domains, including physical and mental health [18]. It has been categorized into the two domains of the Physical Component Summary (PCS) and the Mental Component Summary (MCS). It is a widely used, recognized reliable and validated patient-reported outcome measure, with an internal-consistency reliability of 0.94 for physical functioning and 0.89 for mental functioning [17,19,20,21]. The VR-12 instrument uses five-point ordinal response choices of “no, none of the time”, “yes, a little of the time”, “yes, some of the time”, “yes, most of the time”, and “yes, all of the time”.

#### 2.3.2. Independent Variables

Adults with hyperlipidemia were categorized into the following four mutually exclusive groups: hyperlipidemia only, hyperlipidemia and anxiety, hyperlipidemia and depression, hyperlipidemia and both diseases. We used the ICD-10-CM clinical diagnosis codes from MEPS to identify individuals with depression (‘F34’, ‘F39’, ‘F32’) and anxiety (‘F40’, ‘F41’).

Other independent variables that were evaluated included demographics and socioeconomic variables (gender, age, race/ethnicity, marital status, income, region of residency, education level, employment status, and poverty status), health insurance and medication insurance, perceived physical health, coexisting chronic health conditions, and physical activity.

### 2.4. Statistical Analyses

Baseline characteristics, including mean, standard deviation, frequencies, and percentages were reported using descriptive statistics. Differences in individual characteristics by hyperlipidemia groups were evaluated using chi-square tests. The Kolmogorov–Smirnov test was used to assess that the data were normally distributed before conducting the parametric tests. The variances in HRQoL means between hyperlipidemia groups were determined using ANOVA followed by Tukey’s post hoc test for multiple comparisons to identify which specific groups differed. A multivariable linear regression model was used to evaluate the link between hyperlipidemia groups and HRQoL after adjusting for other independent variables. We considered the complicated survey design of the MEPS in the analysis by adding variance adjustment weights (strata and primary sample unit) from the MEPS with person-level weights. All statistical analysis used the Statistical Analysis System, SAS 9.4 (SAS Institute Inc., Cary, NC, USA).

## 3. Results

### 3.1. Characteristics of the Study Sample

The study sample was composed of 7984 adults with hyperlipidemia; the majority of the study sample were women (55.6%), aged 50 to 64 (75.2%), white (68.4%), and had education higher than high school (89.9%). About 9.0% of adults with hyperlipidemia also experienced depression, 10.2% had anxiety, and 6.8% had both disorders (Table 1).

Women with hyperlipidemia had a significantly higher rates of depression (13.4% vs. 6.0%), anxiety (13.9% vs. 7.3%), and comorbid anxiety and depression (9.7% vs. 4.4%, *p*-value ≤ 0.0001) when compared to men. Furthermore, unemployed hyperlipidemia patients had significantly higher rates of depression (14.3% vs. 9.6%) and comorbid anxiety and depression (11.7% vs. 4.4%, *p*-value < 0.0001) than employed hyperlipidemia patients. Additionally, significantly higher rates of comorbid depression and anxiety were identified among hyperlipidemia adults with heart disease, diabetes, asthma, chronic obstructive pulmonary disease (COPD), and gastroesophageal reflux disease (GERD) than those without these comorbidities (*p*-value < 0.0001).

### 3.2. Health-Related Quality of Life by Hyperlipidemia Groups

**Figure 1:**
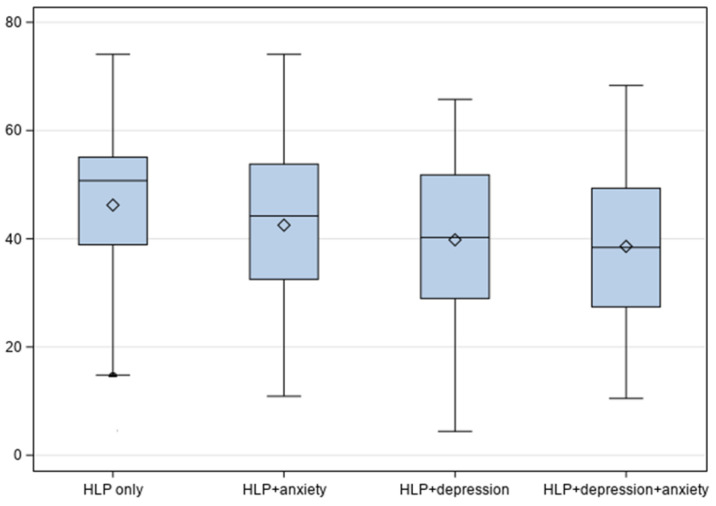
HRQoL Physical Component Summary scores by hyperlipidemic groups.

**Figure 2:**
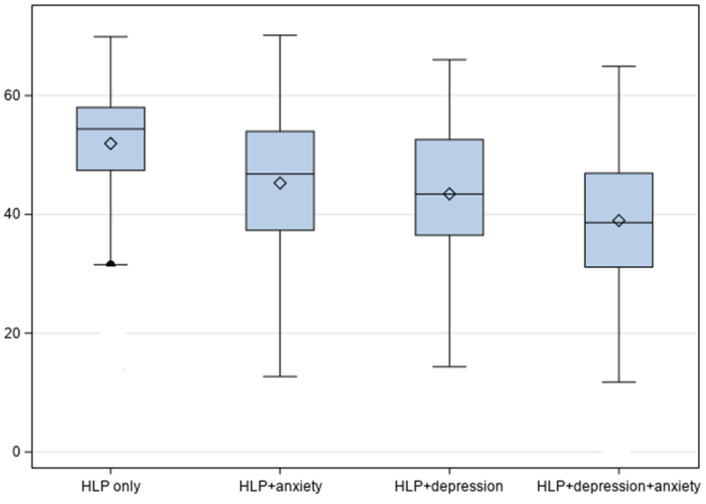
HRQoL Mental Component Summary scores by hyperlipidemic groups.

There were significant differences in the PCS and MCS mean scores between hyperlipidemia groups (Table 2, Figure 1 and Figure 2). For example, compared to other groups, adults with both depression and anxiety had a significantly lower mean PCS score (mean = 40.19, SE = 0.63) and lower mean MCS score (mean = 40.13, SE = 0.62).

Mean difference comparison between different hyperlipidemia groups was performed by using the ANOVA test followed by Tukey’s post hoc test for multiple comparisons (Table 3), which revealed the following:

Patients with only hyperlipidemia have a significant higher mean PCS and MCS scores as compared to the other groups (hyperlipidemia and depression, and hyperlipidemia and anxiety, hyperlipidemia and depression and anxiety).

### 3.3. Adjusted Linear Regression Analysis for Health-Related Quality of Life

The adjusted relationship between hyperlipidemia groups and HRQoL is displayed in Table 4. After adjusting for all confounding variables, hyperlipidemia patients with depression (MCS: β = −5.535, *p*-value < 0.0001), anxiety (MCS: β = −4.406, *p*-value < 0.0001), and both depression and anxiety (MCS: β = −8.730, *p*-value < 0.0001) had a significantly lower HRQoL than those with hyperlipidemia only.

Factors positively associated with HRQoL included employment, perceived general health, and physical activity. For example, adults who perceived their physical health to be excellent or very good have a higher HRQoL in both physical health summary score (PCS: β = 11.709, *p*-value < 0.0001) and mental health summary score (MCS: β = 6.290, *p*-value < 0.0001) compared to those who perceived their health to be poor.

Comorbidities were negatively associated with both PCS and MCS; individuals with heart disease, hypertension, diabetes, asthma, osteoarthritis, and GERD have a lower HRQoL in both physical and mental health summary scores than those without those comorbidities *p*-value < 0.0001).

## 4. Discussion

The results presented in this research provide an insight into the negative relationship between comorbid depression and anxiety and HRQoL in a nationally representative sample of US adults with hyperlipidemia. Four hyperlipidemia groups were compared, as follows: hyperlipidemia only, hyperlipidemia and depression, hyperlipidemia and anxiety, and hyperlipidemia with both depression and anxiety. In terms of mental comorbidities, in this study it was revealed that women with hyperlipidemia had significantly higher rates of depression, anxiety, and comorbid conditions compared to men, which is in line with earlier studies [22] highlighting the potential gender disparity in mental health conditions among hyperlipidemia patients, warranting further investigation into hormonal, psychological, or societal factors contributing to this finding. In fact, gender differences exist not only in mental health but also in clinical medicine, treatment, epidemiology, symptoms, and health outcomes, as evident in above 10,000 published research [23]. Another important finding was that unemployed individuals showed higher rates of depression and comorbid conditions than their employed counterparts. Further, higher rates of anxiety and depression were seen in hyperlipidemic adults with comorbidities (i.e., heart disease, diabetes, asthma, COPD, and GERD); this underscores the bidirectional relationship between physical and mental health, emphasizing the need for integrated care.

In terms of quality of life, our findings highlight the significant impact of depression and anxiety on QoL in individuals with hyperlipidemia by affecting both physical and mental well-being. These results are consistent with a previous study, which has shown that in adults with dyslipidemia, mental health diminishes the overall QoL [15]. Several pathophysiological mechanisms in hyperlipidemia may lead to these mental conditions [9,24,25,26,27]. First, inflammation and oxidative stress induced by high cholesterol levels, which affect brain function and neurotransmitter activity, potentially lead to depression and anxiety; this inflammatory pathway is supported by numerous studies that have linked chronic inflammation to both the onset and progression of depression [25,26,27]. Secondly, cardiovascular risks associated with hyperlipidemia, where fear of events or complications can induce chronic stress and worsen depression and anxiety [27]. Finally, physical limitations, such as those caused by atherosclerosis, which can cause disabilities and fatigue, further contributing to a negative outlook and exacerbating mental health symptoms [9]. The strong link between mental health disorders and reduced HRQoL in hyperlipidemia patients underscores the importance of addressing psychological conditions alongside physical health.

Further, hyperlipidemic adults with chronic comorbidities such as heart disease, hypertension, diabetes, arthritis, asthma, and GERD have significantly reduced HRQoL. In our study sample, approximately two-thirds of adults with hyperlipidemia had hypertension, and one-third had diabetes. The presence of these conditions increases the risk of cardiovascular events and negatively impacts quality of life. Diabetes can lead to complications such as neuropathy and kidney issues, further affecting overall health. Together, hypertension, diabetes, and hyperlipidemia significantly worsen cardiovascular health and contribute to a decline in health-related quality of life (HRQoL). Implementing lifestyle changes, including improvements in diet and exercise, is crucial for managing these conditions. A study in China among 9339 subjects with dyslipidemia reported that a higher healthy lifestyle score (HLS) was based on the following factors: smoking, alcohol drinking, diet, body mass index, and physical activity was linked to a lower risk of hypertension and diabetes, emphasizing the importance of preventative strategies [28]. In addition, exploring the relationship between these comorbidities and HRQoL is vital for improving patient care, and future research should focus on how interventions targeting hypertension and diabetes impact HRQoL in hyperlipidemic patients.

Furthermore, employment, good perceived general health, and physical activity were positively associated with HRQoL; these findings suggest that interventions promoting physical activity and employment could enhance HRQoL. Employment might serve as a protective factor due to better financial security, access to healthcare, and social engagement. The association between employment status and quality of life among adults was investigated using the Korea Health Panel survey data [29]. Exercise has been shown to lower depression and anxiety and it is hypothesized that these mood enhancements result from exercise’s effects on the hypothalamic–pituitary–adrenal axis and the increase in blood flow to the brain [30]. Additionally, exercise is associated with better cardiovascular health and lower cholesterol levels [30,31]. Therefore, tailored interventions targeting at-risk groups (e.g., unemployed individuals, those with comorbidities, and those who are physically inactive) are essential. In addition, enhancing mental health support and integrating it with chronic disease management could improve overall outcomes. Therefore, to effectively manage the complexities of comorbidities, healthcare systems must change to a more integrated and patient-centered approach. Value-based healthcare models, which reward providers based on improvements in HRQoL, can improve patient well-being and reduce healthcare utilization by prioritizing proactive management of hyperlipidemia and comprehensive care [32].

### 4.1. Study Strengths and Limitations

A significant emphasis of the earlier research has been the connection between depression and hyperlipidemia. Thus, this is the first study to evaluate the relationship between depression and anxiety and HRQoL among adults with hyperlipidemia using a nationally representative sample of adults in the US. In this study, several variables (i.e., confounders) were also measured, including sociodemographic characters, health and medication insurance coverage, perceived health, physical activity, and concurrent comorbidities. In this study, there were certain limitations when interpreting this study’s findings. Hyperlipidemia treatment and the severity of their illness were not adjusted in the analysis, which may have a relationship with HRQoL. In addition, MEPS does not cover information on the severity or type of depression or anxiety, which could impact the HRQoL of adults with hyperlipidemia. Finally, in this study, only adults were included; thus, the findings cannot be generalized to the elderly population.

### 4.2. Clinical Practice, Policy, Research, and Public Health Implications

The findings from this study can be used to enhance healthcare services and resource allocation at the healthcare providers’ and policymakers’ levels to minimize the negative consequences of depression and anxiety in individuals with hyperlipidemia and improve public health. In addition, in this study, it is suggested that healthcare providers should, therefore, regularly screen and treat depression and anxiety as early treatment can improve HRQoL and other hyperlipidemia health outcomes. The public health implications from this study call for lifestyle changes as physical activity can significantly improve the severity of their illnesses and their HRQoL. Further studies in this area are necessary as there is a substantial unmet need for additional research to determine the impact of these mental illnesses in HRQoL for patients with hyperlipidemia. In addition, other mental HRQoL instruments should be considered for future studies, such as the Recovering Quality of Life-Utility Index (ReQoL), a patient-reported outcome measure (PROM) that evaluates the quality of life for people with different mental health conditions [33].

In addition, future studies need to evaluate the quality of life in patients with dyslipidemia without mental health problems, since the quality of life is negatively influenced by mental health.

## 5. Conclusions

In this study, valuable insights are provided into the multifaceted factors influencing health outcomes in hyperlipidemia, highlighting the need for a holistic and equitable approach to healthcare delivery. Adults with hyperlipidemia have lower HRQoL due to depression and anxiety in this nationally representative study. These psychological sufferings could impact hyperlipidemia health outcomes. Therefore, it is crucial to prioritize early diagnosis and the treatment of depression and anxiety. Also, this research found the adverse effects of coexisting chronic conditions on HRQoL while highlighting the benefits of employment and regular exercise on HRQoL. The findings from this study have implications for clinical practice and policy to improve healthcare services and resource allocation. Promoting lifestyle changes, specifically physical activity, is essential at the public health level, as it may improve the HRQoL of adults with hyperlipidemia.

## Figures and Tables

**Table 1:** Characteristics of the study sample (*n* = 7984), number, and row percentage of characteristics by hyperlipidemia (HLD) groups.

		Total Sample		HLD Only		HLD and Depression		HLD and Anxiety		HLD and Depression and Anxiety			
		N	Wt.%	N	Wt.%	N	Wt.%	N	Wt.%	N	Wt.%	*p*-Value	Sig.
All		7984	100.0	5800	73.8	787	9.3	787	10.2	610	6.8		
Age in years													
	18–39	522	7.1	354	67.7	37	7.4	74	15.0	57	9.9	0.008	**
	40–49	1308	17.7	948	71.9	114	8.2	136	11.5	110	8.4		
	50–64	6154	75.2	4498	74.8	636	9.7	577	9.4	443	6.1		
Gender													
	Women	3925	44.4	2504	63.0	509	13.4	490	13.9	422	9.7	<0.0001	***
	Men	4059	55.6	3296	82.4	278	6.0	297	7.3	188	4.4		
Race/ethnicity													
	White	4651	68.4	3119	69.6	554	10.8	557	11.8	421	7.9	<0.0001	***
	African American	1238	10.7	983	81.6	94	6.6	85	6.1	76	5.7		
	Latino	1472	12.8	1180	82.6	91	5.5	107	7.6	94	4.3		
	Others	623	8.1	518	85.0	48	6.3	38	6.2	19	2.5		
Marital status													
	Married	4497	62.2	3511	78.2	336	7.7	423	9.5	227	4.6	<0.0001	***
	Widow/Sep/Div.	2220	23.8	1417	66.0	331	13.2	228	10.4	244	10.4		
	Never married	1267	14.0	872	67.3	120	9.6	136	13.2	139	9.9		
Education level													
	< HS	523	4.0	400	77.5	43	9.2	48	7.7	32	5.5	0.257	
	HS	812	7.5	570	70.2	87	9.8	81	10.1	74	9.9		
	> HS	6584	87.9	4780	73.9	651	9.2	652	10.3	501	6.6		
Region													
	Northeast	1291	16.7	949	76.8	106	7.5	110	7.8	126	7.8	0.003	**
	Mid-west	1841	23.3	1250	70.2	230	11.0	199	10.5	162	8.3		
	South	3150	39.6	2302	73.0	282	8.9	340	12.0	226	6.1		
	West	1702	20.4	1299	76.9	169	9.5	138	8.2	96	5.5		
Employment													
	Employed	4916	68.1	3909	79.3	344	6.9	421	9.3	242	4.4	<0.0001	***
	Not employed	3067	31.9	1890	61.9	443	14.3	366	12.1	368	11.7		
Poverty status													
	Poor	1342	11.4	793	56.3	194	15.3	167	13.0	188	15.3	<0.0001	***
	Near Poor	1403	13.8	975	70.7	161	11.1	121	8.3	146	10.0		
	Middle Income	2098	25.0	1546	72.8	189	8.8	231	12.2	132	6.1		
	High Income	3141	49.7	2486	79.1	243	7.6	268	9.0	144	4.2		
Health Insurance													
	Private	5254	74.4	4079	77.5	433	8.0	475	9.6	267	4.8	<0.0001	***
	Public	2401	22.4	1443	60.0	337	13.9	295	12.4	326	13.7		
	Uninsured	329	3.2	278	83.4	17	5.6	17	8.1	17	3.0		
Rx Insurance													
	Rx insurance	4692	67.6	3661	77.6	371	7.8	429	9.8	231	4.7	<0.0001	***
	No Rx insurance	3292	32.4	2139	65.7	416	12.3	358	10.9	379	11.1		
General health													
	Excellent/Very good	2801	39.9	2266	80.9	199	6.6	222	8.9	114	3.6	<0.0001	***
	Good	2941	36.2	2198	74.1	271	9.4	289	10.1	183	6.5		
	Fair/poor	2242	23.9	1336	61.5	317	13.5	276	12.6	313	12.4		
Physical activity													
	3 times/week	3389	44.1	2653	79.1	270	7.0	284	8.7	182	5.2	<0.0001	***
	No exercise	4576	55.6	3134	69.6	517	11.1	499	11.3	426	7.9		
Heart													
	Yes	1134	13.5	744	68.4	156	12.1	128	11.5	106	8.1	0.013	*
	No	6850	86.5	5056	74.6	631	8.8	659	10.0	504	6.5		
Hypertension													
	Yes	4938	58.9	3544	73.1	521	10.0	502	10.2	371	6.7	0.409	
	No	3046	41.1	2256	74.7	266	8.3	285	10.2	239	6.8		
Diabetes													
	Yes	2759	30.9	1925	70.7	322	11.5	245	9.2	267	8.6	<0.0001	***
	No	5225	69.1	3875	75.1	465	8.3	542	10.7	343	5.9		
Asthma													
	Yes	991	11.2	561	58.5	128	12.8	160	16.4	142	12.3	<0.0001	***
	No	6993	88.8	5239	75.7	659	8.8	627	9.4	468	6.1		
COPD													
	Yes	609	6.7	310	49.8	97	17.6	107	17.9	95	14.7	<0.0001	***
	No	7375	93.3	5490	75.5	690	8.7	680	9.6	515	6.2		
Arthritis													
	Yes	1359	15.5	788	60.0	208	14.3	185	14.1	178	11.5	<0.0001	***
	No	6625	84.5	5012	76.3	579	8.3	602	9.5	432	5.9		
GERD													
	Yes	1264	15.0	728	58.7	190	14.4	177	15.4	169	11.5	<0.0001	***
	No	6720	85.0	5072	76.4	597	8.4	610	9.3	441	5.9		

Asterisks (*) represent significant differences in hyperlipidemia groups from chi-square tests, *** *p* < 0.001; ** 0.001 ≤ *p* < 0.01; * 0.01 < *p* < 0.05, COPD: chronic obstructive pulmonary disease; GERD: gastroesophageal reflux disease; HLD: hyperlipidemia; Rx: medication. Sig: significance. Widow/Sep/Div.: widowed, separated, and divorced.

**Table 2:** Health-related quality of life weighted means scores by hyperlipidemia groups.

		Total Sample		Hyperlipidemia Only		Hyperlipidemia and Anxiety		Hyperlipidemia and Depression		Hyperlipidemia and Depression and Anxiety		
		Mean	SD	Mean	SE	Mean	SE	Mean	SE	Mean	SE	*p*-Value
HRQoL												
	PCS	44.63	12.01	47.42	0.24	44.40	0.70	41.25	0.63	40.19	0.63	<0.0001
	MCS	49.44	10.15	52.34	0.15	46.44	0.52	44.59	0.54	40.13	0.62	<0.0001

Mean differences by hyperlipidemia groups were analyzed by ANOVA test. HRQoL: health-related quality of life; MCS: Mental Component Summary; PCS: Physical Component Summary; SE: standard error; SD: standard deviation.

**Table 3:** Post hoc analysis of the mean difference in health-related quality of life between hyperlipidemia groups.

	Physical Component Summary					Mental Component Summary			
HLP Group	Comparison Group	Mean Difference	Confidence Interval		Sig	Mean Difference	Confidence Interval		Sig
HLP only	HLP + ANX	3.70	2.56	4.85	***	6.66	5.76	7.56	***
HLP only	HLP + Dep	6.42	5.27	7.56	***	8.48	7.58	9.38	***
HLP only	HLP + DEP + ANX	7.63	6.35	8.91	***	12.98	11.97	13.99	***
HLP + ANX	HLP only	−3.70	−4.85	−2.56	***	−6.66	−7.56	−5.76	***
HLP + ANX	HLP + DEP	2.71	1.20	4.23	***	1.82	0.62	3.01	***
HLP + ANX	HLP + DEP + ANX	3.92	2.30	5.55	***	6.31	5.04	7.59	***
HLP + DEP	HLP only	−6.42	−7.56	−5.27	***	−8.48	−9.38	−7.58	***
HLP + DEP	HLP + ANX	−2.71	−4.23	−1.20	***	−1.82	−3.01	−0.62	***
HLP + DEP	HLP + DEP + ANX	1.21	−0.41	2.83		4.49	3.22	5.77	***
HLP + DEP + ANX	HLP only	−7.63	−8.91	−6.35	***	−12.98	−13.99	−11.97	***
HLP + DEP + ANX	HLP + ANX	−3.92	−5.55	−2.30	***	−6.31	−7.59	−5.04	***
HLP + DEP + ANX	HLP + DEP	−1.21	−2.83	0.41		−4.49	−5.77	−3.22	***

ANX: anxiety; DEP: depression; HLP: hyperlipidemia; Sig: significant level, *** *p* < 0.001.

**Table 4:** Adjusted multivariable linear regressions on HRQoL.

		Health-Related Quality of Life							
		Physical Component Summary				Mental Component Summary			
		β	SE	95% CI	Sig.	β	SE	95% CI	Sig.
Hyperlipidemia (HLP) groups									
	HLP and depression	−1.23	0.045	−1.19–−1.26	***	−5.53	0.019	−5.50–−5.57	***
	HLP and anxiety	−0.05	0.022	−0.49–−0.06	**	−4.40	0.031	−4.38–−4.42	***
	HLP and depression and anxiety	−0.73	0.075	−0.69–−0.75	***	−8.73	0.133	−8.71–−8.74	***
	HLP only (Ref.)								
Age in years									
	18–39	2.64	0.017	2.45–2.68	***	−1.96	0.030	−1.94–−1.99	***
	40–49	1.50	0.025	1.48–1.52	***	−1.33	0.033	−1.31–−1.38	***
	50–64 (Ref.)								
Gender									
	Women	0.55	0.022	0.54–0.58	***	0.49	0.020	0.42–0.51	***
	Men (Ref.)								
Race/ethnicity									
	African Am	0.21	0.048	0.20–0.25	***	0.42	0.031	0.39–0.47	***
	Latino	1.64	0.026	1.62–1.66	***	0.38	0.031	0.34–0.39	***
	Others	0.18	0.021	0.15–0.21	***	−0.29	0.046	−0.22–−0.31	***
	White (Ref.)								
Marital status									
	Married	−0.32	0.022	−0.29–−0.35	***	1.11	0.03	1.10–1.14	***
	Widow/Sep/Div.	0.03	0.028	0.01–0.06		1.19	0.05	1.15–1.23	***
	Never married (Ref.)								
Education level									
	>HS	−0.96	0.030	−0.94–−0.99	***	1.12	0.024	1.10–1.16	***
	HS	−0.93	0.045	−0.93–−0.98	***	1.35	0.035	1.30–1.38	***
	<HS (Ref.)								
Region									
	Northeast	1.09	0.011	1.05–1.12	***	−0.12	0.019	−0.10–−0.15	***
	Mid-west	0.37	0.008	0.33–0.39	***	0.13	0.016	0.11–0.17	***
	South	−0.34	0.026	−0.32–−0.37	***	0.09	0.038	0.05–0.12	*
	West (Ref.)								
Employment									
	Employed	3.71	0.050	3.47–3.79	***	1.52	0.031	1.50–1.57	***
	Not employed (Ref.)								
Poverty status									
	Poor	−1.72	0.033	−1.70–−1.75	***	−1.62	0.029	−1.59–−1.67	***
	Near Poor	−1.49	0.023	−1.46–−1.51	***	−0.80	0.044	−0.78–−0.84	***
	Middle Income	−0.76	0.021	−0.73–−0.79	***	0.28	0.022	0.25–0.31	***
	High Income (Ref.)								
Health Insurance									
	Private	−0.16	0.071	−0.13–−0.18	*	−0.66	0.099	−0.61–−0.73	***
	Public	−3.35	0.061	−3.30–−3.39	***	−1.73	0.105	−1.70–−1.77	***
	Uninsured (Ref.)								
Rx Insurance									
	Rx insurance	−0.33	0.022	−0.31–−0.38	***	−0.82	0.046	−0.79–−0.86	***
	No Rx insurance (Ref.)								
General health									
	Excellent/very good	11.70	0.039	10.95–11.8	***	6.29	0.034	6.10–6.32	***
	Good	7.26	0.020	7.10–7.32	***	4.42	0.035	4.39–4.47	***
	Fair/poor (Ref.)								
Physical activity									
	3/week	1.60	0.015	1.57–1.66	***	0.96	0.027	0.93–0.98	***
	No exercise (Ref.)								
Heart									
	Yes	−2.78	0.040	−2.56–−2.90	***	−0.22	0.084	−0.20–−0.19	**
Hypertension									
	Yes	−1.20	0.017	−1.18–−1.28	***	0.16	0.030	0.13–0.18	***
Diabetes									
	Yes	−1.37	0.029	−1.33–−1.33	***	0.48	0.024	0.44–0.53	***
Asthma									
	Yes	−1.81	0.031	−1.78–−1.85	***	−1.03	0.023	−1.00–−1.09	***
COPD									
	Yes	−1.71	0.036	−1.65–−1.76	***	0.74	0.047	0.56–0.79	***
Arthritis									
	Yes	−3.32	0.051	−3.30–−3.41	***	−0.25	0.024	−0.21–−0.29	***
GERD									
	Yes	−1.08	0.034	−1.06–−1.10	***	−0.56	0.057	−0.50–−0.60	***

Asterisks denote statistical significance in parameter estimates from adjusted multivariable linear regressions on health-related quality of life. *** *p* < 0.001; ** 0.001 ≤ *p* < 0.01; * 0.01 < *p* < 0.05, COPD: chronic obstructive pulmonary disease; GERD: gastroesophageal reflux disease; Rx: medication; Ref: reference group; SE: standard error; Sig: significance. Widow./Sep./Div.: widowed, separated, and divorced.

## Data Availability

Researchers can access the publicly accessible dataset used in this study from the MEPS database at this URL: https://meps.ahrq.gov/data_stats/download_data_files.jsp (accessed on 12 November 2024).

## List of Abbreviations

HRQoL: health-related quality of life, MCS: mental health component score, PCS: physical health component score.
